# The effect of *Fucus vesiculosus*, an edible brown seaweed, upon menstrual cycle length and hormonal status in three pre-menopausal women: a case report

**DOI:** 10.1186/1472-6882-4-10

**Published:** 2004-08-04

**Authors:** Christine F Skibola

**Affiliations:** 1 School of Public Health, Molecular Epidemiology and Toxicology Laboratory, University of California, Berkeley, CA 94720, USA

## Abstract

**Background:**

Rates of estrogen-dependent cancers are among the highest in Western countries and lower in the East. These variations may be attributable to differences in dietary exposures such as higher seaweed consumption among Asian populations. The edible brown kelp, *Fucus vesiculosus *(bladderwrack), as well as other brown kelp species, lower plasma cholesterol levels. Since cholesterol is a precursor to sex hormone biosynthesis, kelp consumption may alter circulating sex hormone levels and menstrual cycling patterns. In particular, dietary kelp may be beneficial to women with or at high risk for estrogen-dependent diseases. To test this, bladderwrack was administered to three pre-menopausal women with abnormal menstrual cycling patterns and/or menstrual-related disease histories.

**Case Presentation:**

Intake of bladderwrack was associated with significant increases in menstrual cycle lengths, ranging from an increase of 5.5 to 14 days. In addition, hormone measurements ascertained for one woman revealed significant anti-estrogenic and progestagenic effects following kelp administration. Mean baseline 17β-estradiol levels were reduced from 626 ± 91 to 164 ± 30 pg/ml (*P *= 0.04) following 700 mg/d, which decreased further to 92.5.0 ± 3.5pg/ml (*P *= 0.03) with the1.4 g/d dose. Mean baseline progesterone levels rose from 0.58 ± 0.14 to 8.4 ± 2.6 ng/ml with the 700 mg/d dose (*P *= 0.1), which increased further to 16.8 ± 0.7 ng/ml with the 1.4 g/d dose (*P *= 0.002).

**Conclusions:**

These pilot data suggest that dietary bladderwrack may prolong the length of the menstrual cycle and exert anti-estrogenic effects in pre-menopausal women. Further, these studies also suggest that seaweed may be another important dietary component apart from soy that is responsible for the reduced risk of estrogen-related cancers observed in Japanese populations. However, these studies will need to be performed in well-controlled clinical trials to confirm these preliminary findings.

## Background

Epidemiological studies show that incidence rates of estrogen-dependent diseases such as cancers of the breast, endometrium and ovary are among the highest in Western, industrialized countries, while rates are much lower in China and Japan [1,2]. These disparities may be attributable, in part, to differences in dietary and environmental exposures associated with affluent and modern lifestyles that promote estrogenic stimulation and hormone imbalances [3-5]. Although the mechanisms are not fully understood, epidemiological and experimental data suggest that exposure to estrogens, through endogenous production and exogenous exposures resulting in an imbalance in the estrogen/progesterone ratio, may be the most critical determinants in disease risk [6-8]. In estrogen-sensitive tissues, estrogen triggers cell proliferation, and through prolonged stimulation, hyperplasia [9] and possibly neoplasia can occur. Reproductive factors associated with increased exposure to menstruation resulting in persistent and sustained estrogenic stimulation, such as shorter menstrual cycles, reduced parity, early menarche, and late menopause, are known to increase risk of endometriosis and estrogen-dependent cancers [10,11], while post-menopausal obesity, hormone replacement therapy and alcohol consumption may be associated with increased breast cancer risk [12-14]. Therefore, limiting exposure to estrogens and reducing the overall number of menstrual cycles in one's lifetime through dietary and lifestyle changes may be the simplest means to reduce disease risk. In particular, the identification of dietary compounds that have estrogen- reducing effects holds great promise in developing chemopreventive strategies to abrogate risk of these diseases.

Studies show that Japanese women have longer menstrual cycle lengths (greater than the 28 day average) and lower circulating estrogen levels compared to Western populations [15-17], which until now has been at least partly attributed to the increased intake of soy protein among Asian populations [18-20]. Another less explored component but main staple of the Japanese diet is seaweed, which accounts for approximately 10–25% of their food intake [21,22]. Other reported estimated daily intakes are as high as 3–13 g/day [23]. A major source of dietary seaweed among Japanese populations is the edible brown kelp, wakame (*Undaria pinnatifida*) and kombu (*Laminaria japonica*). These species and the Atlantic brown kelp, bladderwrack (*Fucus vesiculosus)*, have been shown to exert powerful anti-hypertensive activity related to angiotensin-I-converting enzyme inhibition [24], to possess antibacterial and antioxidant properties related to their high polyphenolic content [25], and to prevent dioxin absorption and accelerate dioxin excretion in rats [26]. Other chemopreventive properties such as antiviral activity [27,28], immunostimulatory effects [29], anti-proliferative effects on 7,12-dimethylbenz(a)-anthracene-induced rat mammary tumors [30,31], and anti-tumor and anti-metastatic activities in xenograft mouse models [32], have been associated with the high level of sulfated polysaccharides, also known as fucoidans, found in brown seaweed.

Intake of bladderwrack, as well as other brown kelp species, also has been shown to alter cholesterol metabolism and to significantly lower plasma cholesterol levels [33,34]. A possible mechanism of action involves competitive inhibition by fucosterols found in kelp. Since cholesterol is the precursor involved in steroid hormone biosynthesis, a reduction in cholesterol bioavailability could lower circulating plasma 17β-estradiol levels that may lead to alterations in menstrual cycling patterns in pre-menopausal women. Until now, no studies have been performed in humans to determine the effects of brown kelp on menstrual cycling patterns and sex hormone status in pre-menopausal women, particularly in women with or at risk for estrogen-dependent diseases. To explore the hypothesis that kelp consumption could reduce circulating17β-estradiol levels and attenuate menstrual cycle irregularities, bladderwrack was administered to three pre-menopausal women with abnormal menstrual cycling patterns and/or menstrual-related disease histories.

## Case presentation

Three pre-menopausal women with abnormal menstrual cycling histories volunteered for the present study. An abnormal menstrual cycle was defined as one or more of the following: menstrual cycles of <26 or >32 days in length; menstrual cycles consisting of >8 menstruating days; or anovulatory menstrual cycling. Study subject characteristics are outlined in Table 1. Subject 1 had a history of hypermenorrhea (excessive blood loss during menstruation), polymenorrhea (shorter than average menstrual cycle length of 28 days), anovulatory menstrual cycles, and was diagnosed with luteal phase deficiency and endometriosis (through laparoscopy). Subject 2 suffered from hypermenorrhea and polymenorrhea. Subject 3 suffered from hypermenorrhea and was diagnosed with endometriosis. All three women reported a history of dysmenorrhea (painful menses). Otherwise, all women were in general good health and free of any chronic diseases. All women were active and exercised approximately three times per week. No hormones or other medications were taken for >3 months prior to the inception of the study. No soy protein products were consumed during the study period.

**Table 1:** Study Subject Characteristics

**Subject**	**Age**	**Body weight (lb)**	**Menstrual cycle history**	**Medical conditions/health status**	**Medications**
1	43	142	hypermenorrhea, polymenorrhea, dysmenorrhea, luteal phase deficiency	endometriosis; otherwise healthy	none
2	42	138	hypermenorrhea, polymenorrhea, dysmenorrhea	general good health	none
3	21	126	hypermenorrhea, dysmenorrhea	endometriosis; otherwise healthy	none

The protocol was approved by the Committee for the Protection of Human Subjects of the University of California at Berkeley. The nature of the study was explained, and written informed consent was obtained from all study subjects.

### Source and dose of bladderwrack (Fucus vesiculosus)

Dried, powdered bladderwrack was obtained from Maine Coast Sea Vegetables (Franklin, ME) and encapsulated in 350 mg capsules. Two capsules were administered daily for the low dose treatment (700 mg) and four capsules were administered daily for the high dose treatment (1.4 g). Bladderwrack dosage levels were chosen to fall within the range of reported dietary seaweed intakes (10–25%) of the total diet reported for Japanese populations [21,22]. This was calculated by assuming a total 500 g/d total dietary intake and a range between 50–125 g/d (wet weight) or 0.5–1.25 g/d (dry weight) seaweed intake. This calculation falls below the estimated 3–13 g/d intake reported by Teas et al. [23].

### Experimental protocols

Details of the study protocol are outlined in Table 2. All women provided self-reported menstrual cycling histories for the three months prior to the treatment period. In addition, 17β-estradiol and progesterone serum measurements were taken for Subject 1 throughout the course of the study as outlined in Table 2. Ovulation was monitored through body basal temperature. Since the average length of her cycle was 16 days prior to treatment and she was not ovulating at the inception of the study, baseline hormone levels were ascertained on a set day (menstrual cycle day 12) for two consecutive cycles prior to the administration of 700 mg/d bladderwrack for two additional cycles. During the treatment period, serum hormone levels were measured on days 12 and 21 for the first cycle (which was another anovulatory cycle) and on day 21 thereafter during the treatment period. Subjects 2 and 3 were administered 700 mg/d of bladderwrack beginning on day 21 of their menstrual cycles and followed for two consecutive cycles. Subsequently, Subjects 1 and 3 agreed to continue the experiment for two additional cycles at which time they received a daily dose of 1.4 g/d kelp. Menstrual cycling logs were maintained on all subjects during the entire course of the experiment. Subjects were monitored at least weekly to insure compliance to the supplement regimen.

**Table 2:** Treatment protocol and timeline

**Subject**	**Pretreatment menstrual cycling history obtained**	**Pretreatment serum 17β-estradiol and progesterone levels ascertained (Cycle and day)**	**Treatment period/dose**	**Treatment serum 17β-estradiol and progesterone levels ascertained (Cycle and day)**	**Treatment period/dose**	**Treatment serum 17β-estradiol and progesterone levels ascertained (Cycle and day)**
1	Cycles 1–3	Cycle 2, day 12; Cycle 3, day 12	Cycle 4–5/ 700 mg/d	Cycle 4, day 12; Cycle 4, day 21; Cycle 5, day 21	Cycle 6–7/ 1.4 g/d	Cycle 6, day 21; Cycle 7, day 21
2	Cycles 1–3	NA	Cycle 4–5/ 700 mg/d	NA	NA	NA
3	Cycles 1–3	NA	Cycle 4–5/ 700 mg/d	NA	Cycle 6–7/ 1.4 g/d	NA

### Hormone assays

All hormone assays were performed by Quest Laboratories (San Diego, CA), an outside-certified clinical laboratory. Serum 17β-estradiol and progesterone levels were measured in duplicate by radioimmunoassays. Blank and control sera were run with each assay. The coefficient of variation (a measure of laboratory error) was consistently low (<15%) for 17β-estradiol and progesterone.

### Statistical methods

Statistical analyses were performed by unpaired t-tests (2-sided) with a commercially available statistical software package (Stata, College Station, Texas). All statistical tests were considered significant for p ≤ 0.05. Results are referred to as borderline significant for 0.05 < p ≤ 0.10.

### Clinical findings

There were no adverse side effects reported and bladderwrack was well tolerated by all three women.

### Effects of treatment on length of menstrual cycle and total days of menstruation

**Figure 1:**
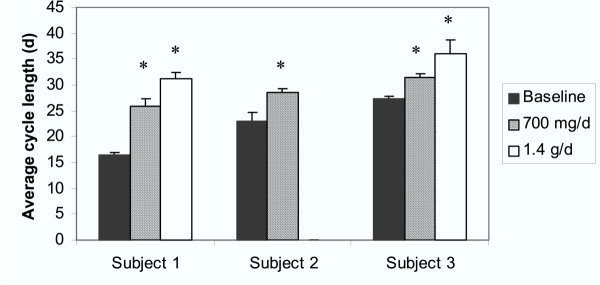
Average menstrual cycle length in days for Subjects 1–3 at baseline (black bars) and following bladderwrack administration of 700 mg/d (diagonal striped bars) and 1.4 g/d (white bars). Black bars indicate the averages of 3 menstrual cycles; diagonal striped and white bars indicate the averages of 2 menstrual cycles; and whiskers indicate standard deviations. * *P *value <0.05.

Following treatment, all women exhibited a significant increase in menstrual cycle lengths (Figure 1). Specifically, in Subject 1, who had a 30-year history of irregular menses, the menstrual cycle length increased from an average of 16.3 ± 0.6 days to 26.0 ± 1.4 days with the low dose treatment (*P *< 0.002), which increased by approximately 5 additional days to 31.2 ± 1.1 days following administration of the higher dose (*P *< 0.001). In Subject 2, the average cycle length increased 5.5 days, from 23.0 ± 1.7 to 28.5 ± 0.7 days (*P *= 0.03). Subject 3 exhibited a 4-day increase in menstrual cycle length from 27.3 ± 0.6 to 31.5 ± 0.7 days with the 700 mg dose (*P *= 0.005) that increased by approximately 6 more days to 36.0 ± 2.8 days with the 1.4 g dose (*P *= 0.01).

**Figure 2:**
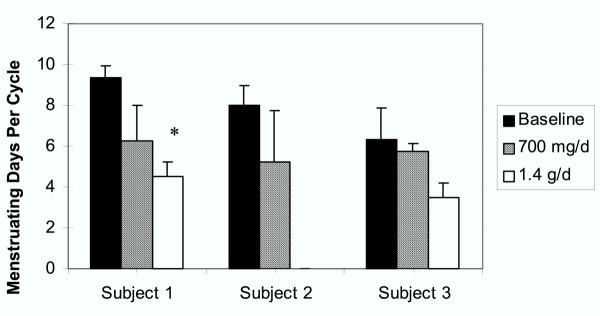
Average number of days of menstruation per cycle for Subjects 1–3 at baseline (black bars) and following bladderwrack administration of 700 mg/d (diagonal striped bars) and 1.4 g/d (white bars). Each bar indicates averages from two menstrual cycles; whiskers indicate standard deviations. * *P *value <0.05.

Along with increased menstrual cycle lengths, all women reported marked reductions in blood flow and average number of days of menstruation following bladderwrack treatment (Figure 2). Subject 1 reported the most significant reduction in total days of menstruation, changing from an average 9.3 ± 0.6 to 6.3 ± 1.8 days (*P *= 0.06) with the low dose and to 4.5 ± 0.7 average days with the high dose (*P *< 0.003). Subject 2, who only took the low dose, also experienced a marked reduction in number of days of menstruation, from 8.0 ± 1.0 to 5.3 ± 2.5 days (*P *= 0.06). Subject 3 exhibited a decrease in total menstruating days averaging from 6.3 ± 1.5 to 5.8 ± 0.4 days (*P *= 0.65) with the low dose, and to 3.5 ± 0.7 days (*P *= 0.10) with the 1.4 g/d dose. Subjects 1 and 3, who both suffered from endometriosis, reported substantial alleviation from pain during menstruation and throughout the menstrual cycle following bladderwrack treatment.

Subject 1 also reported an ovulatory cycle following 2 months on the 700 mg/d kelp intervention, and continued to have ovulatory cycles while on the 1.4 g/d dose.

### Effects of treatment on serum estradiol and progesterone levels

A significant anti-estrogenic and progestagenic dose response was observed in plasma estradiol and progesterone levels in Subject 1 (Table 3). Specifically, the mean baseline 17β-estradiol levels were reduced from 626 ± 91 to 164 ± 30 pg/ml (*P *= 0.04) with the low dose (700 mg/d), which decreased further to 92.5 ± 3.5 pg/ml (*P *= 0.03) with the higher dose (1.4 g/d). Furthermore, mean baseline progesterone level rose from 0.58 ± 0.14 to 8.4 ± 2.6 ng/ml with the lower 700 mg/d dose (*P *= 0.1), which increased further to 16.8 ± 0.7 ng/ml with the 1.4 g/d dose (*P *= 0.002).

**Table 3:** Average circulating plasma 17β-estradiol and progesterone levels prior to and during kelp intervention for Subject 1

	**Pre-treatment baseline levels**	**700 mg/d dose**	***P*-value**	**1.4 g/d dose**	**P-value**
17β-estradiol (pg/ml)	626 ± 91	164 ± 30	0.04	92.5 ± 3.5	0.03
Progesterone (ng/ml)	0.58 ± 0.14	8.4 ± 2.6	0.10	16.8 ± 0.7	0.002

All measures are from 2 month averages ± S.D.

### Discussion of clinical findings

The results of this preliminary pilot study suggest that bladderwrack consumption can effectively increase the length of the menstrual cycle and reduce the total number of days of menstruation in pre-menopausal women. These effects were most marked in the two women that had shorter than average cycles (16 and 23 days) versus the normal range of 26 to 32 days seen in women in Western populations. Menstrual cycles were further lengthened with increasing dose, which may suggest a linear dose-response effect. However, since there was not a sufficient washout period between the 700 and 1400 mg/d doses, an effect of length of time of dosing rather than a dose response effect cannot be ruled out. Nonetheless, these marked increases in menstrual cycle length may have beneficial health effects in lowering risk of estrogen-dependent diseases such as endometriosis and ovarian, endometrial, and breast cancers as reported in a number of studies [16,35-38]. Menstrual characteristics are surrogate markers that may reflect a woman's overall exposure to and production of endogenous hormones. Shorter menstrual cycle lengths and prolonged menstruation confer longer follicular and luteal phases where estrogen and progesterone levels and endometrial and breast cell proliferation rates are at their highest. A nearly fourfold increase in mitotic activity in the breast lobules occurs during the luteal phase of the menstrual cycle [39], while the highest proliferation rates (nearly 100-fold) in the endometrium occur during the follicular phase [40]. Therefore, fewer menstrual cycles over a woman's lifetime would decrease the amount of time during which the breast and endometrial epithelia would be exposed to high levels of proliferation, which may decrease overall disease risk.

Bladderwrack consumption also led to a marked reduction in circulating 17β-estradiol levels and an increase in progesterone levels in a subject who exhibited high serum estrogen levels and progesterone deficiency prior to the intervention. While estrogen's proliferative effects on mammary gland development and endometrial and breast tumorigenesis are well documented, progesterone's role in these processes is not as well defined. Studies show that progesterone deficiency is associated with increased endometrial cancer incidence [41], and that progesterone inhibits estrogen-induced luminal epithelial proliferation in the uterus [42]. However, progesterone has been shown to both stimulate and inhibit the growth of experimental mammary tumors [43], and the use of synthetic progestins in hormone replacement therapy has been associated with an increased risk of breast cancer [43]. Experimental rat models have elucidated progesterone's vital role in pregnancy-induced morphological changes in the breast, which confer protection against breast cancer [44]. Further, epidemiological studies suggest that it is not pregnancy alone but early first parity and increasing number of pregnancies that are associated with reduced breast cancer risk [45,46]. These studies suggest that the effects of progesterone in breast cancer risk may be dependent on timing and the type and level of progesterone/progestin exposure. Thus, the anti-estrogenic/progestagenic activity of kelp observed in this study warrants further investigation in its role in breast cancer and other hormone-dependent diseases.

### Study limitations

Due to the small number of subjects and the lack of a control group, this study will need to be repeated in a larger, randomized population of women with placebo controls. Other weaknesses of the present study are the lack of data on luteinizing hormone and follicular stimulating hormone levels which would provide pertinent information regarding the effects of dietary bladderwrack on ovulation and the luteal and follicular phases of the menstrual cycle. The potential beneficial impact that dietary bladderwrack may have on abrogating symptoms of endometriosis warrants a closer look at a larger population of women suffering from this disease. However, studies should also be performed in women with normal menstrual cycles who have sex hormone levels within clinically normal ranges to determine the impact of dietary kelp on menstrual cycling patterns and hormone levels in the general population.

## Conclusions

The observed responses to bladderwrack consumption in this study suggest that dietary modification may lead to significant changes in the regulation of the menstrual cycle by increasing the length of the cycle, stimulating ovulation, and lowering the estrogen/progesterone ratio in pre-menopausal women. Such changes may be beneficial particularly with regard to women at high risk of estrogen-dependent diseases or who are experiencing fertility problems. Results from these preliminary experiments also suggest that bladderwrack administration may alleviate hypermenorrhea and dysmenorrhea, which may provide some relief in the treatment of endometriosis. Although these reported effects are generally in a beneficial direction, their clinical significance is yet to be determined in a well-controlled study.

## Future Directions

The critical role of hormones in breast, endometrial, and ovarian cancers in women and prostate cancer in men has long been recognized. Given the vast rise of these cancers in the U.S. and our limited success with prevention and treatment, there is clearly a need for the identification of novel, non-cytotoxic chemopreventive agents. Future investigations should clarify the role of bladderwrack and other seaweed species on estrogen and progesterone metabolism, to evaluate its potential binding affinity to estrogen and progesterone receptors, and to determine its effects on proliferation in hormone-sensitive tissues. These investigations should also be expanded to include effects of bladderwrack on other sex hormones including the androgens and gonadotropins. In this regard, animal and *in vitro *studies are currently underway in our laboratory to elucidate the potential mechanisms and clinical relevance of bladderwrack bioactivity, and to identify and isolate the active components involved.

## Competing Interests

None declared.

## Pre-publication history

The pre-publication history for this paper can be accessed here:


